# Searching for Factors Raising the Incidence of Metabolic Syndrome Among 45-60-Year-Old Women

**DOI:** 10.14336/AD.2017.1027

**Published:** 2018-10-01

**Authors:** Szkup Małgorzata, Brodowski Jacek, Owczarek Aleksander Jerzy, Choręza Piotr, Jurczak Anna, Grochans Elżbieta

**Affiliations:** ^1^Department of Nursing, Pomeranian Medical University in Szczecin, Szczecin, Poland; ^2^Primary Care Department, Pomeranian Medical University in Szczecin, Szczecin, Poland; ^3^Department of Statistics, Department of Instrumental Analysis, School of Pharmacy with the Division of Laboratory Medicine in Sosnowiec, Medical University of Silesia, Katowice, Poland; ^4^Department of Clinical Nursing, Pomeranian Medical University in Szczecin, Szczecin, Poland

**Keywords:** Metabolic Syndrome X, Fat Mass and Obesity Associated (FTO) Protein, Melanocortin Melanocortin 4 Receptor, Peroxisome Proliferator-Activated Receptor gamma

## Abstract

Metabolic syndrome is an increasing health problem, whose pathogenesis may be associated with genetic factors. The main purpose of our study was to assess relationships between MetS and the presence of the *FTO* rs9939609, the *MC4R* rs17782313, and the *PPAR-γ* rs1801282 polymorphisms in 45-60-year-old women. The study included patients from the general population of the Westpomeranian Province (Poland). The mean age was 54.3 ± 4.2 years. The research procedure involved taking structured history, physical examination, anthropometric measurements, and collecting blood for biochemical and genetic analysis. The patients who met the diagnostic criteria for MetS constituted 38.35% of all participants (sample size: 425 patients). The comparison of blood biochemical parameters revealed numerous differences between the women with MetS and those from the control group. Genetic analysis demonstrated that the T allele of the *FTO* gene was a factor substantially decreasing the incidence of MetS in the study sample (ORT vs. A = 0.734; 95% CI: 0.555 - 0.970; p < 0.05). Other polymorphisms were not directly related to MetS incidence. Conclusions: 1. MetS-related abnormalities are widespread in the population of 45-60-year-old Polish women. Those most common are the elevated serum total cholesterol and LDL levels, increased insulin resistance and BMI scores, as well as visceral obesity. 2. No direct relationships were demonstrated between MetS and the gene polymorphisms analyzed in our study except for the *FTO* rs9939609, whose A allele and A/A genotype seemed to predispose to metabolic disorders.

Metabolic syndrome (MetS) — whose incidence in the world population is steadily growing — is a huge challenge for the public health sector [1]. Metabolic disorders, regarded as the components of MetS, substantially contribute to the incidence of and mortality for acute coronary syndrome (2.5 higher risk), cerebral stroke and major adverse cardiac events (twice higher risk), and increase the total death rate by 1.5 times [2, 3].

Since the diagnostic criteria change over time, it is difficult to compare epidemiological data concerning the widespread of this disorder. Nevertheless, the percentage of people showing MetS symptoms is very high [4].

According to estimates, in 2011-2012 this health problem affected 34.7% of the general U.S. population with a predominance of elderly people [5]. In Poland, MetS is observed in 20.3% of the adult population, however its incidence depends on sex (considerably more women than men) and age (7.5% of individuals between 18 and 39 years of age, 23.9% of those between 40 and 59 years of age, and 39.5% of those over 60) [3]. Research conducted in Poland indicated also visible area-related differences: the highest estimated percentage of women with this disorder was found in the Westpomeranian Province (25%), where our research was carried out, and Opolskie Province (26%) [6]. There is no available clear and updated information on the incidence of this disorder depending on sex. The epidemiological situation is subject to dynamic changes. Therefore, one of the goals of our study was to assess the incidence of MetS among women between the ages of 45-60 years.

Risk factors include dyslipidemia (elevated levels of triglycerides and decreased levels of high-density lipoprotein cholesterol), visceral abdominal obesity, as well as increased blood pressure and fasting glucose. MetS is defined as the coexistence of at least three out of five factors indicating a higher metabolic and cardiovascular risk [7]. The amount of adipose tissue (especially accumulated in the abdominal area), and modification of its functions influence the course of metabolic processes and homeostasis in the whole body. The role of abdominal adiposity is so important that when in 2005 the International Diabetes Federation proposed MetS definition — based on the criteria included in the Third Report of the National Cholesterol Education Program Expert Panel on Detection, Evaluation, and Treatment of High Blood Cholesterol in Adults Treatment Panel III — it deemed abdominal obesity (identified on the basis of waist size measurement) as a necessary condition for a diagnosis of MetS [8]. Some authors questioned an arbitrary assumption that MetS can only occur in abdominally obese people. In 2009, it was finally decided that there should not be any obligatory criteria for MetS diagnosis, however the emphasis was put to the usefulness of waist size measurements performed as a part of screening tests [7].

A lot of evidence suggests that metabolic changes occurring in the organism as a result of aging favor MetS development [9]. Hence, in our study we focused on people at especially high risk from MetS, namely 45-60-year-old women.

Though behavioral factors, such as smoking, a bad diet, and lack of physical activity are regarded as important cofactors for MetS [10], some role is also attributed to genetic factors, which — as research outcomes show — are responsible for about 10-30% of MetS cases [11, 12]. Genetic factors potentially contributing to MetS are polymorphic forms of genes that have effects on the expression of MetS-related abnormalities, such as obesity, lipid and carbohydrate metabolism disorders, and insulin resistance [13]. In the study presented in this article, we analyzed the influence of selected factors on the incidence of MetS in the population of Polish women.

The *FTO* gene is located in chromosome region 16q12.2. It consists of nine exons that span over 400kb [14]. The influence of the *FTO* gene on a human organism has not yet been fully understood. It is assumed that the mechanism of its activity can be associated with a higher demand for energy drawn from food, a feeling of satiety, and the role it plays in adipogenesis [15, 16, 17]. Out of several *FTO* variants known so far, the most commonly described is the *rs*9939609 T>A polymorphism. As has been noticed, it entails higher mortality rate and metabolic disorders, irrespective of whether a person is obese or not. It is regarded as an independent risk factor for obesity and faster biological aging in a group of non-obese people [18]. The relationship between MetS and the above-mentioned polymorphism is not clear, and findings reported by various authors stand in contradiction to each other [19, 20, 21, 22].

The* MC4R* gene regulates metabolism through its influence on dietary habits. Its rs17782313 variant has been demonstrated to be related to obesity in the Asian population and the Caucasian race [23]. There is a strong connection between this SNP and obesity in all age groups, however the minor alleles of this SNP are typically more strongly associated with higher BMIs than the major alleles [24, 25]. Nevertheless, the relationship between the *MC4R* rs17782313 and the risk of MetS has not been unambiguously confirmed. Available results lead to contradictory conclusions [26, 27, 28, 29].

The *PPAR-γ* gene is located on the short arm of the third chromosome (3p25), and includes nine exons that span over >100kb [30]. *PPAR*-γ is responsible for regulation of adipogenesis, sensitivity to insulin, and fat metabolism. It also modulates immunity and inflammation [31]. Disorders of the *PPAR-γ* function entail various pathologies, such as type 2 diabetes, glomerulitis, atherosclerosis, and pulmonary arterial hypertension (PAH) [32]. Out of two most important isoforms of the receptor (*PPAR-γ1* and *PPAR-γ2*), we focused on *PPAR-γ2* due to the fact that its expression is observed almost exclusively in adipocytes [30].

Several *PPAR-γ* variants have been identified so far, of which the most common is the *Pro12Ala* (*rs*1801282) polymorphism, being the effect of the mutation in codon 12 of exon B of the *PPAR-γ2* isoform. This mutation is a result of the substitution of guanine for cytidine, and consequently the change of proline to alanine (CCA-GCA) [33]. The connection between MetS and the *Pro12Ala* polymorphism has not yet been clearly explained, and available reports provide contradictory conclusions [34, 35, 36, 37].

MetS is a vital public health problem, having effects on the quality of human life and increasing the risk of untimely death. The development of MetS is determined by the interaction of numerous environmental lifestyle-related factors. Its etiology may also be underlain by genetic predisposition associated with the presence of the *FTO* rs9939609, the *MC4R* rs17782313, and the *PPAR-γ* rs1801282 polymorphisms.

The aim of our study was to identify factors contributing to MetS and to assess its incidence among 45-60-year-old women in the Westpomeranian Province (Poland) with regard to:

- biochemical and anthropometric parameters (the serum levels of insulin, glucose, total cholesterol, low-density lipoprotein cholesterol (LDL), high-density lipoprotein cholesterol (HDL), triglycerides (TG), and C-reactive protein (CRP) on an empty stomach; the HOMA-IR index, non-HDL cholesterol, body mass index (BMI), waist to hip ratio (WHR); body weight, hip size, waist size, systolic (sRR) and diastolic (dRR) blood pressure, menstruation, smoking, diagnosed coronary heart disease, type 2 diabetes, and hyperlipidemia).
- genetic factors (the *FTO* rs9939609, the *MC4R* rs17782313, and the *PPAR-γ* rs1801282 polymorphisms).

## MATERIALS AND METHODS

This original, cross-sectional representative study involved 425 women, aged 45-60 years, from the general population of the Westpomeranian Province (Poland). A quota sampling method was applied. According to the Westpomeranian Province Statistical Yearbook from 2015, the group of women aged 45-59 years in the area analyzed in our study included 179565 subjects [38]. Using the sample size calculator, and assuming the confidence interval to be 95%, the estimated fraction size — 0.5, and the maximum error — 5%, we established that the study sample should consist of at least 383 individuals plus 10 percent drop-out.

All subjects gave their informed consent for inclusion in the study. The study was conducted in accordance with the Declaration of Helsinki, and the protocol was approved by the Bioethical Commission of the [covered for blind review] (permission numbers KB-0012/181/13 and KB-0012/104/11).

Recruitment was performed based on advertisement in local papers, and information posters placed on bulletin boards in public places (offices, shops, workplaces, primary care outpatient clinics). The criteria for inclusion in the study were sex, age bracket, and written consent to take part in it. The criteria for temporary exclusion from the study were current oncological and psychiatric problems, as well as thyroid diseases. From the study sample we excluded 24 subjects who reported the aforementioned diseases during medical history taking; 17 more individuals were excluded from further analysis as they failed to answer all the questions in the structured medical history, and 4 others due to a pre-laboratory error.

The participants were divided into two groups. The first group included 162 women with MetS diagnosed if they had at least three out of five MetS components in accordance with the modified criteria proposed by the International Diabetes Federation (IDF) in 2009. These components were: waist size ≥ 80 cm, fasting glycemia ≥ 100 mg/dl or pharmacotherapy for hyperglycemia, the level of triglycerides ≥ 150 mg/dl or related pharmacotherapy, HDL level < 50 mg/dl or related pharmacotherapy, and increased blood pressure (systolic ≥ 130 and/or diastolic ≥ 85 mmHg) or pharmacotherapy for hypertension [7]. The control group comprised of 263 women without MetS.

### Description of the research procedure

The first stage of the study involved taking structured medical history from each patient. The participants were asked about their sociodemographic data (education, place of residence, marital status, professional activity) and health status (menstruation, smoking, coronary heart disease, type 2 diabetes, hyperlipidemia). Next, physical examination was performed, and anthropometric measurements were taken. We assessed body weight, height, hip size, waist size, and blood pressure.

At the next stage of the study, ulnar venous blood was taken from each patient using the Vacutainer system. The blood was collected by qualified and experienced nurses. The blood was collected in the treatment room, and then delivered to the laboratory in accordance with the relevant rules and procedures. The levels of insulin, total cholesterol, HDL, LDL, TG, glucose, and CRP were determined in blood serum. Next, the following indices were calculated:

- the level of non-HDL cholesterol calculated by subtracting the level of HDL cholesterol from the level of total cholesterol (mg/dl);
- the homeostasis model of assessment-insulin resistance (HOMA-IR) index, calculated according to the formula: serum glucose level (mg/dl) x insulin level (μIU/ml) divided by 405 [39].
- body mass index (BMI) calculated according to the formula: BMI = kg/m^2^;
- waist-to-hip ratio (WHR).

The results of our study, compared to the laboratory norms, are shown in Table 1.

As the last stage of our study, we performed genetic analysis of three gene polymorphisms: *FTO rs*9939609, *MC4R rs*17782313, and *PPAR-γ rs*1801283.

**Table 1:** Characteristics of the biochemical and anthropometric parameters of the women with regard to the normal ranges.

	Reference range	Below the norm [n (%)]	The norm [n (%)]	Above the norm [n (%)]
**Insulin** [uIU/ml]	2.6 — 24.9 uIU/ml	3 (0.71%)	399 (93.88%)	23 (5.41%)
**Total cholesterol** [mg/dl]	115 — 190 mg/dl	1 (0.23%)	105 (24.71%)	319 (75.06%)
**HDL** [mg/dl]	> 50 mg/dl	110 (25.94%)	314 (74.06%)	
**LDL** [mg/dl]	< 115 mg/dl		135 (31.76%)	290 (68.24%)
**TG** [mg/dl]	< 150 mg/dl		332 (78.12%)	93 (21.88%)
**Non-HDL** [mg/dl]	< 145 mg/dl		177 (41.65%)	248 (58.35%)
**Glucose** [mg/dl]	60 — 99 mg/dl		321 (75.53%)	104 (24.47%)
**CRP** [mg/l]	0.0 — 5.0 mg/l		378 (88.94%)	47 (11.06%)
**HOMA-IR**	≤ 2.5 [36]		269 (63.29%)	156 (36.71%)
**sRR** [mmHg}	≤ 130 mmHg		199 (46.82%)	226 (53.18%)
**dRR** [mmHg}	≤ 85 mmHg		298 (70.12%)	127 (29.88%)
**Waist size** [cm]	≤ 80 cm [3]		115 (27.06%)	310 (72.94%)
	**Underweight** **[n** (%)]	**Norm** **[n** (%)]	**Overweight** [n **(%)]**	**Obesity** **[n (%)]**
**BMI** (refernce range: 18.5 - 24.99 kg/m^2^)	2 (0.47%)	135 (31.91%)	162 (38.30%)	126 (29.79%)
	**Gynoid fat distribution** **WHR < 0.8 [n (%)]**		**Android fat distribution** **WHR ≥ 0.8 [n (%)]**	
**WHR**	79 (18.59%)		(346) 81.41%	

n — number of case

### Genotyping of the FTO rs9939609 (T>A), the MC4R rs17782313 (T>C), and the PPAR-γ rs1801282 (C>G) polymorphisms

Genomic DNA was isolated from whole blood according to standard procedures. All genotyping was performed with the fluorescence resonance energy transfer method real time using the Light Cycler System 1.0 (Roche Diagnostic, Poland). The following conditions were applied for the polymorphism in the genes. Polymerase chain reaction (PCR) was performed with 50 ng DNA in a total volume of 20 ml containing 2 ml reaction mix, 0.5 mM of each primer, 0.2 mM of each hybridization probe and 2 mM MgCl2 according to the manufacturer’s instructions for 35 cycles of denaturation (95°C for 10min), annealing 60°C for 10 seconds) and extension (72°C for 15 seconds). After amplification, a melting curve was generated by holding the reaction at 40°C for 20 seconds and then heating slowly to 85°C. The fluorescence signal was plotted against temperature to give melting curves for each sample.

The polymorphisms were determined on the basis of analysis of the melting curves. In the *PPAR-γ rs*1801282 (C>G) polymorphism, peaks were obtained at 53.14^0^C for the G allele and at 62.12^0^ C for the C allele. In the *FTO rs*9939609 (T>A) polymorphism, peaks were obtained at 58.02 ^0^C for the A allele and at 63.08 ^0^C for the T allele. The fluorescence signal was plotted against temperature to give melting curves for each sample. Peaks were obtained at 49.5°C for the T allele and at 58.23°C for the C allele.

### Statistical analysis

Statistical analysis was performed using STATISTICA 10.0 PL (StatSoft, Cracow, Poland,) and R environment. Statistical significance was set at a p value below 0.05. All tests were two-tailed. Nominal and ordinal data were expressed as percentages, whilst interval data were expressed as a mean value ± standard deviation in the case of normal distribution, or as median (lower quartile-upper quartile) in the case of data with skewed or non-normal distribution. Distribution of variables was evaluated by the Shapiro-Wilk test and homogeneity of variances was assessed by the Levene test. Two groups (with and without MetS) were compared either with the t-Student test in case of normal data distribution or after logarithmic transformation, or with the Mann-Whitney U test in case of heavy-skewed data distribution. Nominal data were compared with χ^2^ or Fisher exact test. In order to assess the relationship between MetS and genotypes, four inheritance models were tested with Bayesian Information Criterion used to choose the best model (the smallest one). Odds ratio with confidence interval was used to show the influence of variables and alleles on MetS occurrence.

**Table 2:** Comparative analysis of particular biochemical and anthropometric parameters, and blood pressure in the women with regard to MetS.

	MetS (+) (n = 162)	MetS (-) (n = 263)	p
**Insulin** [uIU/ml]	12.89 (9.26 — 17.20)	8.70 (6.30 — 10.30)	< 0.001
**Fasting glycemia** [mg/dl]	101.0 (90.0 — 117.0)	84.0 (78.0 — 91.0)	< 0.001
**HOMA-IR**	4.741 (2.240 — 4.447)	1.790 (1.260 — 2.334)	< 0.001
**Total cholesterol** [mg/dl]	219.0 ± 52.3	218.1 ± 36.7	0.858
**LDL** [mg/dl]	133.4 ± 46.6	132.1 ± 34.4	0.761
**HDL** [mg/dl]	55.3 ± 16.7	67.8 ± 15.4	< 0.001
**TG** [mg/dl]	147.0 (103.0 — 194.0)	84.0 (67.0 — 109.0)	< 0.001
**Non-HDL** [mg/dl]	164.2 ± 51.4	150.3 ± 37.5	< 0.01
**CRP** [mg/dl]	1.5 (1.1 - 2.8)	2.4 (1.3 - 3.8)	< 0.001
**Body mass** [kg]	77.5 ± 13.0	71.0 ± 13.0	< 0.001
**BMI** [kg/m^2^]	28.93 (25.80 — 33.20)	25.80 (23.40 — 29.00)	< 0.001
**Hip size** [cm]	103.9 (98.6 — 109.0)	100.4 (96.0 — 105.9)	< 0.001
**Waist size** [cm]	91.4 ± 10.7	85.5 ± 10.0	< 0.001
**WHR**	0.872 ± 0.073	0.841 ± 0.073	< 0.001
s**RR** [mmHg]	135.5 ± 14.6	122.2 ± 14.3	< 0.001
**dRR** [mmHg]	81.8 ± 9.6	76.9 ± 10.3	< 0.001

n — number of cases; mean ± standard deviation; median (lower quartile - upper quartile); p — significance level

## RESULTS

The mean age was 54.3 ± 4.2 years. 44.94% of the women had secondary and 39.53% had higher education, 66.12% lived in cities with a population of over 100 000, 71.29% were married, and 74.35% were professionally active. We collected data concerning the women’s health functioning: 16.47% of the participants were menstruating women, 20.23% smoked cigarettes, 4.23% had coronary heart disease, 13.17% had type 2 diabetes, and 14.35% had hyperlipidemia.

Analysis, whose purpose was to characterize the study sample in terms of biological parameters with reference to the normal ranges, demonstrated that many of the women had lipid metabolism disorders, namely increased levels of total cholesterol, LDL cholesterol, and non-HDL cholesterol. Other big problems were body build abnormalities (obesity, visceral obesity, and overweight) and the HOMA-IR suggesting higher insulin resistance. The android fat distribution was the most often observed (Table 1).

**Table 3:** Comparative analysis of particular elements of the women’s health functioning with regard to MetS.

	MetS (+) (n = 162) [n (%)]	MetS (-) (n = 263) [n (%)]	p
**Menstruation** [N (%)]	18 (11.11%)	52 (19.77%)	< 0.05
**Smoking** [N (%)]	32 (19.75%)	52 (19.77%)	0.870
**Coronary heart disease** [N (%)]	16 (9.88%)	2 (0.76%)	< 0.001
**Type 2 diabetes** [N (%)]	49 (30.25%)	7 (2.66%)	< 0.001
**Hyperlipidemia** [N (%)]	51 (31.48%)	10 (3.80%)	< 0.001

n — number of cases; p — significance level

The study sample was divided into two groups: the study group consisting of the women who met the diagnostic criteria for MetS (n = 162), and the control group comprised of the women without this health problem (N = 263). The comparison of both groups in terms of particular parameters revealed large statistically significant differences in the levels of insulin, fasting glycemia, HDL, TG, non-HDL cholesterol, and CRP, as well as the HOMA-IR index, body mass, BMI, waist size, hip size, WHR, and systolic and diastolic blood pressure. Such differences were not found only in the case of total cholesterol and LDL cholesterol, whose levels were only slightly higher in the women who met the diagnostic criteria for MetS (Table 2).

Analysis of other factors associated with health functioning revealed statistically significant differences between the groups in terms of the percentage of menstruating women. Our results show that menstruation plays an important part in the development of MetS: the menopausal women met the criteria for MetS almost twice as often as their menstruating counterparts (OR = 1.992; 95% CI: 1.118 - 3.546; p < 0.05). What is more, coronary heart disease (OR was not calculated due to a small size of the subgroup), type 2 diabetes (OR = 15.858; 95% CI: 6.968 — 36.090, p < 0.001), and hyperlipidemia (OR = 11.624; 95% CI: 5.694 — 23.730; p < 0.001) were noticeably more common in the group with MetS than in the control group (Table 3).

In the whole study sample, the A/T genotype was the most frequent of the *FTO* variants (46.59%). The A/A genotype was considerably more common in the group with MetS (25.31% vs. 15.97%), while the T/T genotype seemed to protect against MetS. These relationships showed a tendency to be statistically significant (p = 0.056). The T allele of the *FTO* gene was a factor visibly reducing the incidence of MetS in the study sample (ORT vs. A = 0.734; 95% CI: 0.555 - 0.970; p < 0.05).

Analysis of the *MC4R* genotype distribution demonstrated that the T/T genotype was the gene variant that predominated (64.47%) in both groups. There were no statistically significant differences in the distribution of the genotypes and alleles between the groups.

The C/C genotype was the most common of the *PPAR-γ* variants (69.27%) in the whole study sample, and the difference in its frequency between both groups was very small (66.05% vs. 71.26%). The distribution of other genotypes of this gene (C/G and G/G), and the C and G alleles was similar. The differences between the groups were not statistically significant (Table 4).

**Table 4:** Analysis of the distribution of the *FTO* rs9939609, the *MC4R* rs17782313, and the *PPAR-γ* rs1801282 polymorphisms with regard to MetS.

	FTO genotype			FTO allele	
	A/A n (%)	A/T n (%)	T/T n (%)	A allele n (%)	T allele n (%)
**MetS** (+)	41 (25.31%)	72 (44.44%)	49 (30.25%)	154 (42.3%)	170 (35.0%)
**MetS** (-)	42 (15.97%)	126 (47.91%)	95 (36.12%)	210 (57.7%)	316 (65.0%)
∑	83 (19.53%)	198 (46.59%)	144 (33.88%)		
**p**	p = 0.056			p < 0.05	
	**MC4R genotype**			**MC4R allele**	
	**C/C** **n (%)**	**C/T** **n (%)**	**T/T** **n (%)**	**C allele** **n (%)**	**T allele** **n (%)**
**MetS** (+)	2 (1.23%)	58 (35.80%)	102 (60.69%)	62 (38.5%)	262 (38.0%)
**MetS** (-)	8 (3.04%)	83 (31.56%)	172 (65.40%)	99 (61.5%)	427 (62.0%)
∑	10 (2.35%)	141 (33.18%)	274 (64.47%)		
**p**	p = 0.363			p = 0.909	
	**PPAR genotype**			**PPAR allele**	
	**C/C** **n (%)**	**C/G** **n (%)**	**G/G** **n (%)**	**C allele** **n (%)**	**G allele** **n (%)**
**MetS** (+)	107 (66.05%)	47 (29.01%)	8 (4.94%)	261 (37.7%)	63 (40.9%)
**MetS** (-)	186 (71.26%)	59 (22.61%)	16 (6.13%)	431 (62.3%)	91 (59.1%)
∑	293 (69.27%)	106 (25.06%)	24 (5.67%)		
**p**	p = 0.320			p = 0.461	

n — number of cases; ∑ — sum of cases; p — significance level

At the last stage of the study, we tested standard inheritance models: co-dominant, dominant, recessive, and over-dominant. In the case of the *PPAR-γ* and the *MC4R* polymorphisms, statistically significant relationships were not demonstrated in any of the tested models. Analysis of the *FTO* polymorphism inheritance models showed that in the recessive model, the incidence of MetS among the A/A genotype carriers was higher than among those with the T/T-A/T genotype (Table 5). This was also the best model according to the BIC criterion.

## DISCUSSION

Due to its increasing incidence in the world population, MetS has been a subject of growing interest over the past two decades. In our study, the percentage of women who met the criteria for MetS was alarmingly high (38.35%). Our findings confirmed unquestionably that MetS involves negative changes in the biochemical composition of blood (serum insulin, glucose, HDL, and TG levels), worse results of anthropometric measurements (higher body mass, BMI, hip size, waist size, WHR), and increased — both systolic and diastolic — blood pressure. Thus, it contributes to the widespread of the most serious diseases of civilization (coronary heart disease, type 2 diabetes, hyperlipidemia) and a higher death rate [40].

**Table 5:** Odds ratios (OR) for the relationships between MetS and the *FTO* rs9939609, the *MC4R* rs17782313, and the *PPAR-γ* rs1801282 SNPs calculated assuming different models of inheritance

Model	Genotype	MetS (+)	MetS (-)	OR (95% CI)	p-value	BIC
Association between *PPAR-γ* and MetS (n = 423, crude analysis)							
**Condominant**	C/C	107 (66.0%)	186 (71.3%)	1.00	0.32	578.9
	C/G	47 (29.0%)	59 (22.6%)	1.38 (0.88 - 2.17)		
	G/G	8 (4.9%)	16 (6.1%)	0.87 (0.36 - 2.10)		
**Dominant**	C/C	107 (66.0%)	186 (71.3%)	1.00	0.26	573.8
	C/G-G/G	55 (34.0%)	75 (28.7%)	1.27 (0.84 - 1.94)		
**Recessive**	C/C-C/G	154 (95.1%)	245 (93.9%)	1.00	0.60	574.8
	G/G	8 (4.9%)	16 (6.1%)	0.80 (0.33 - 1.90)		
**Overdominant**	C/C-G/G	115 (71.0%)	202 (77.4%)	1.00	0.14	572.9
	C/G	47 (29.0%)	59 (22.6%)	1.40 (0.90 - 2.19)		
Association between *FTO* and MetS (n = 425, crude analysis)							
**Condominant**	T/T-A/T	49 (30.2%)	95 (36.1%)	1.00	0.06	577.4
	A/T	72 (44.4%)	126 (47.9%)	1.11 (0.71-1.74)		
	A/A	41 (25.3%)	42 (16%)	1.89 (1.09-3.28)		
**Dominant**	T/T	49 (30.2%)	95 (36.1%)	1.00	0.21	575.5
	A/T-A/A	113 (69.8%)	168 (63.9%)	1.30 (0.86-1.98)		
**Recessive**	T/T-A/T	121 (74.7%)	221 (84%)	1.00	< 0.05	571.6
	A/A	41 (25.3%)	42 (16%)	1.78 (1.10-2.89)		
**Overdominant**	T/T-A/A	90 (55.6%)	137 (52.1%)	1.00	0.49	576.6
	A/T	72 (44.4%)	126 (47.9%)	0.87 (0.59-1.29)		
Association between *MC4R* and MetS (n = 425, crude analysis)							
**Condominant**	T/T	102 (63%)	172 (65.4%)	1.00	0.34	580.9
	C/T	58 (35.8%)	83 (31.6%)	1.18 (0.78-1.79)		
	C/C	2 (1.2%)	8 (3%)	0.42 (0.09-2.02)		
**Dominant**	T/T	102 (63%)	172 (65.4%)	1.00	0.61	576.8
	C/T-C/C	60 (37%)	91 (34.6%)	1.11 (0.74-1.67)		
**Recessive**	T/T-C/T	160 (98.8%)	255 (97%)	1.00	0.21	575.5
	C/C	2 (1.2%)	8 (3%)	0.40 (0.08-1.90)		
**Overdominant**	T/T-C/C	104 (64.2%)	180 (68.4%)	1.00	0.37	576.2
	C/T	58 (35.8%)	83 (31.6%)	1.21 (0.80-1.83)		

Scientists’ interest in MetS has improved the awareness of the complex integrative physiology. Aside from environmental and behavioral factors, the development of metabolic disorders can also be underlain by genetic determinants. Studies on the *FTO* rs9939609, the *MC4R* rs17782313, and the *PPAR-γ* rs1801282 polymorphisms conducted so far, focused mainly on searching for the relationships between these gene variants and symptoms of metabolic disorders (obesity, insulin resistance, hypertension, and hyperlipidemia). We looked for the connection between the above-mentioned gene variants and MetS as a disorder diagnosed on the basis of the modified IDF criteria from 2009 in the population of 45-60-year-old women in the Westpomeranian Province (Poland).

Our findings show that MetS incidence is related to the presence of the A/A genotype of the *FTO* rs9939609 polymorphism, while the T allele protects against this disorder.

The majority of studies carried out by other authors suggest that there is a relationship between the *FTO* rs9939609 polymorphism and selected components of MetS. The study of 685 Tunisians demonstrated that the *FTO* haplotypes noticeably affected their blood pressure, serum TG level, and fasting glycemia. The authors regarded it as an evidence that *FTO* may play an extremely important part in the development of MetS among Tunisians [41]. Another study conducted among 1967 individuals of both sexes in Turkey revealed that minor alleles were an independent risk factor for obesity in women and for MetS in men [42]. An interesting research was carried out among non-Caucasian geographical ancestries, including the groups of Canadians of South Asian and Chinese descent, Oji-Cree (Ontario, Canada), and Inuit from Greenland. The A allele of the *FTO* rs9939609 polymorphism was found to significantly raise the risk of MetS (OR = 1.23, 95% CI: 1.01 - 1.50; p < 0.05). This tendency was especially apparent in men [43]. The hepatic manifestation of MetS is non-alcoholic fatty liver disease (NAFLD) [44, 45]. The study of 1027 Chinese children demonstrated an effect of the A allele on BMI, and thus its contribution to the increased risk of NAFLD (OR = 1.43; p < 0.05) [46].

In our study, direct relationships between particular genotypes or alleles of the *MC4R* rs17782313 polymorphism and the incidence of MetS were not observed. Similar conclusions were drawn by numerous researchers, who, however, reported that the *MC4R* rs17782313 polymorphism was related to some MetS components. In the Europeans, this gene polymorphism contributed to obesity, fat mass content, and body weight at the population level [47]. The research conducted among Koreans showed that the C allele increased BMI by approximately 0.22 kg/m^2^ [48]. Other studies of the same population revealed association between the *MC4R* C allele and food energy intake, which was regarded as a factor directly related to an elevated risk of obesity [49]. Higher BMI scores were observed in Chinese people and Tatar women, regardless of whether they were homo- or heterozygotes of the C allele of the *MC4R* rs17782313 polymorphism (unlike carriers of the T allele) [50, 51]. The study of Guan et al. did not provide evidence for the relationships between NAFLD and the tested gene polymorphisms [46].

In the study of Polish postmenopausal women, the presence of the T allele of the *MC4R* rs17782313 polymorphism was associated with considerably lower incidence of lipid metabolism disorders. Thus, it seems to be an important protective factor against MetS [52].

The research carried out by Chen et al. among Uyghurs demonstrated that the presence of the C/C genotype of the *PPAR-γ* rs1801282 polymorphism was a stronger contributor to higher systolic blood pressure than the C/G and G/G genotypes [53]. Similar conclusions were also drawn by other authors interested in the influence of this polymorphism on the Chinese population. Nevertheless, direct relationships between the genotypes of this gene and MetS were found neither in Uyghurs nor in Kazakhs [54, 55]. The study of hospital patients in Bosnia and Herzegovina did not demonstrate direct relationships between the mentioned genotypes and MetS, but provided evidence that they involved the risk of developing type 2 diabetes and obesity [56]. The results of meta-analysis performed by Sahebkar did not confirm associations between the modes of inheritance of the tested polymorphisms and the NAFLD [57].

Available analyses show undeniably that mechanisms underlying MetS are complex and determined by numerous factors. From among etiological causes of MetS, we cannot exclude racial and geographic differences. In the study presented here, we assessed the possibility of genetic influence on MetS pathogenesis among Polish women in hopes that our observations will help us understand the molecular etiology of MetS. Seeking genetic determinants of MetS can be an interesting contribution to the knowledge of this increasing health problem. The outcomes of the study described in this article add an essential biological component to the picture of environmental etiological factors and allow us to estimate the strength of the influence of the tested genetic variants on the development of MetS in 45-60-year-old women.

### Limitations

The study sample was recruited through the distribution of information about the possibility of taking part in the study in the local environment, by means of information posters placed on bulletin boards in public places. Despite our efforts, there is a risk that the study sample was not representative, which could affect the results.

To determine insulin resistance, we used the HOMA-IR index, which is a very good but still a surrogate marker. There is great variability in the threshold HOMA-IR levels. A discussion is hold on the HOMA-IR cut-off points for insulin resistance [58]. The cut-off point assumed in our study was 2.5 as recommended in literature [39].

### Conclusions

MetS-related abnormalities are very common in the population of 45-60-year-old women in the Westpomeranian Province (Poland). They are more widespread than one might expect on the basis of available epidemiological data, and those most frequently observed include elevated serum total and LDL cholesterol levels, insulin resistance, visceral obesity, and increased BMI.

MetS diagnosed in 45-60-year-old women noticeably affects their biological status, both in terms of biochemical and anthropometric characteristics.

There were no direct relationships between MetS and the presence of the *PPAR-γ* rs1801282 or the *MC4R* rs17782313 polymorphisms in our study. The only exception was the *FTO* rs9939609, whose A allele and A/A genotype seemed to predispose to metabolic disorders. This relationship was confirmed in the recessive inheritance model.
